# Uncommon synchronous histopathological features of a radicular cyst: a case report

**DOI:** 10.4076/1757-1626-2-9067

**Published:** 2009-08-25

**Authors:** Panagiotis Kafas, Sotirios Kalfas, Tahwinder Upile, Waseem Jerjes

**Affiliations:** 1 Department of Oral Surgery and Radiology, School of Dentistry, Aristotle University, Thessalonica, Greece; 2 Department of Preventive Dentistry, Periodontology and Implant Biology, School of Dentistry, Aristotle University, Thessalonica, Greece; 3 Department of Surgery, University College London Medical School, UK; 4 Unit of Oral and Maxillofacial Surgery, UCL Eastman Dental Institute, UK; 5 Head and Neck Centre, University College London Hospital, UK

## Abstract

Radicular cysts are the most common inflammatory odontogenic cystic lesions. It originates from epithelial residues in periodontal ligaments secondary to inflammation. The pathogenesis involves the activation of epithelial cell rests of Malaseez after physical, chemical or bacterial injury. Microscopically, the cyst is thin with smooth or corrugated inner surface. The most common epithelial lining is stratified squamous; with Rushton's hyaline bodies in 10% of the reported cases. Slow accumulation and deposition of cholesterol during the inflammatory process leads to the formation of "clefts" with acute and chronic inflammatory cells in the proliferating epithelium and connective tissue, respectively. The presence of hemosiderin usually indicates a previous micro-hemorrhage event. While the presence of lipid-laden macrophages or foam cells indicate the presence of cholesterol-removing mechanism from the lesion. We report a rare case of 38-year-old Mediterranean female presented with throbbing right maxillary pain. The diagnosis of radicular cyst was confirmed by the presence of atrophic non-keratinized stratified squamous epithelium. The radicular cyst was associated with hemosiderin, foam cells, subepithelial fibrosis and root canal dystrophic calcification. They represent uncommon synchronous histopathological features.

## Introduction

Radicular cysts are the most common inflammatory odontogenic cystic lesions. It originates from epithelial residues in periodontal ligaments secondary to inflammation. Commonly found at root apices of involved teeth or lateral to the root. These cysts may persist even after removal of the problematic tooth; hence called residual cysts [1-3].

Nearly two thirds of the reported cases were in males in the 4^th ^& 5^th ^decades of life. All teeth-baring areas are susceptible to radicular cyst formation; but more than half of the reported cases were in the maxillary anterior segment [1-3].

Most of the small radicular cysts are asymptomatic and usually discovered during routine dental screening. Larger cysts cause swelling and bony expansion followed by erosion and fluctuation of the overlying soft tissue; this is usually associated with pain and infection with a discharging sinus. Radiographically, they appear as round or ovoid radiolucent areas surrounded by a narrow radio-opaque margin, extending from the lamina dura of the involved tooth [1-3].

The pathogenesis involves the activation of epithelial cell rests of Malassez after physical, chemical or bacterial injury. Three phases of cystic formation has been described: initiation, cyst formation and cyst enlargement [1-3]. This condition is always found to be associated with difficult mechanical and chemical endodontic procedures due to blockage of the root canal [4]. Reactionary dentine may induce production of calcified tissues in order to protect vitality of the tooth [5]. But in case of chronic irritation, direct microorganism invasion leads to pulp necrosis. Actinomyces is the most commonly occurring organism in an infected radicular cyst [1-3].

Histopathologically, the cyst is thin with smooth or corrugated inner surface. The most common epithelial lining is stratified squamous; with Rushton's hyaline bodies in 10% of the reported cases. Slow accumulation and deposition of cholesterol during the inflammatory process leads to the formation of "clefts" with acute and chronic inflammatory cells in the proliferating epithelium and connective tissue, respectively. The fibrous capsule is composed mainly of condensed parallel bundles of collagen fibres peripherally and a loose connective tissue adjacent to epithelial lining. Other reported structures include satellite microcysts, calcifications, mast cells and remnants of odontogenic epithelium [1-3]. The process of haem catabolism may be considered as an appropriate host response; where lysosomes deform the structure of haem into catabolic subparts [6]. The presence of hemosiderin usually indicates a previous micro-hemorrhage event. While the presence of lipid-laden macrophages or foam cells indicate the presence of cholesterol-removing mechanism from the lesion [7]. This mechanism has been described in atherosclerotic patients suffering from ischaemic heart disease [8], but has not been evaluated in radicular cyst lamina propria.

We report a rare case of radicular cyst associated with hemosiderin, foam cells, subepithelial fibrosis and root canal dystrophic calcification.

## Case presentation

A 38-year-old Mediterranean female presented with throbbing right maxillary pain. Clinical examination revealed the presence of oral fistula in the upper right lateral incisor area. The tooth was severely decayed (unrestorable) and tender to percussion. The patient reported remarkable medical history and no allergies.

**Figure 1:**
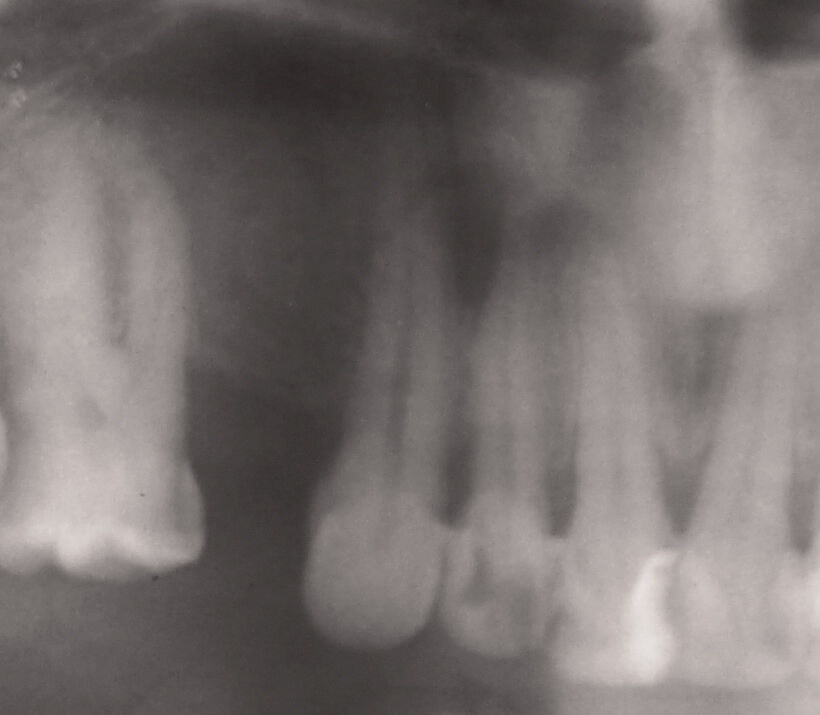
**Dental panoramic tomography showing periapical lesion associated with upper right lateral incisor**.

Radiological evaluation revealed a periapical radiolucent lesion of upper right lateral incisor (Figure 1). Surgical extraction and enucleation of the cyst took place under local anesthesia. The cyst measured 6 mm in diameter.

Histopathological processing involved the immersion of the specimen in buffered formalin for 24 hours, followed by decalcification in EDTA (for hard tissue processing). A microtome was used to cut the specimen into 5 μm blocks and the paraffin sections were stained with hematoxylin and eosin (H&E) prior to histopathological examination.

**Figure 2:**
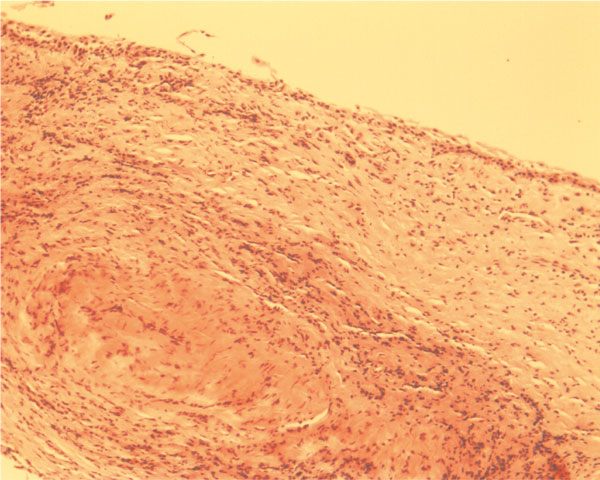
**Subepithelial fibrosis of radicular cyst surrounded by multiple inflammatory cells (H&E ×10)**.

**Figure 3:**
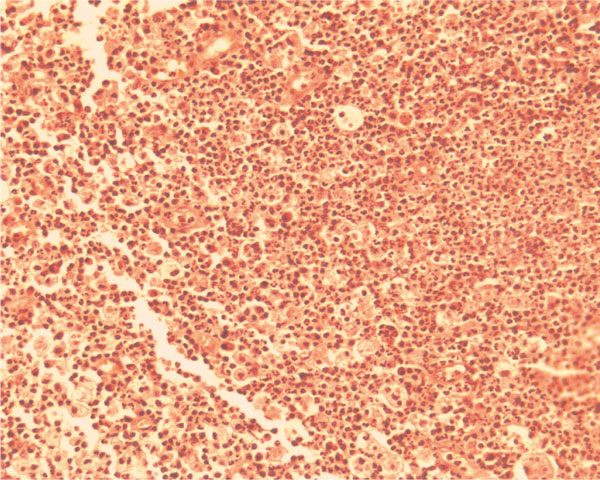
**Lipid-laden macrophages or foam cells expanded in size due to engulfment of necrotic lipid debris. **The presence of PMN's indicates the possible acute exacerbation of the lesion (H&E ×10).

The diagnosis of radicular cyst was confirmed by the presence of atrophic non-keratinized stratified squamous epithelium. In the lamina propria, an oval fibrotic island was observed centrally, which was surrounded by multiple inflammatory cells (Figure 2). In other areas lipid-laden macrophages expressed the presence of foam cells (Figure 3).

**Figure 4:**
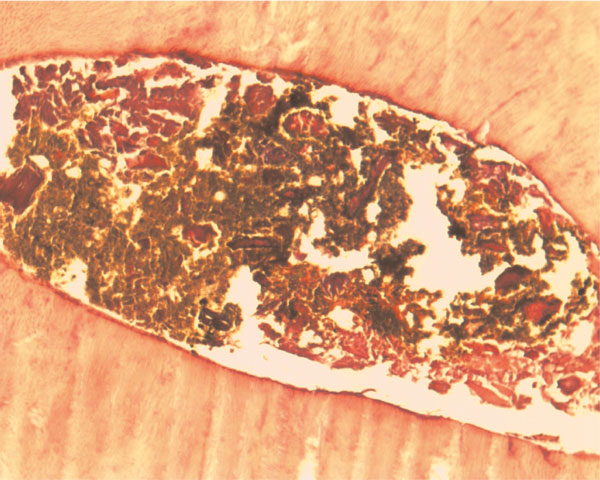
**Liquefaction areas of necrosis due to dystrophic calcification of the obstructed root canal contents**. The brownish material may be a complex of bacterial clouds, necrotic organic content and haem catabolic products (H&E ×20).

**Figure 5:**
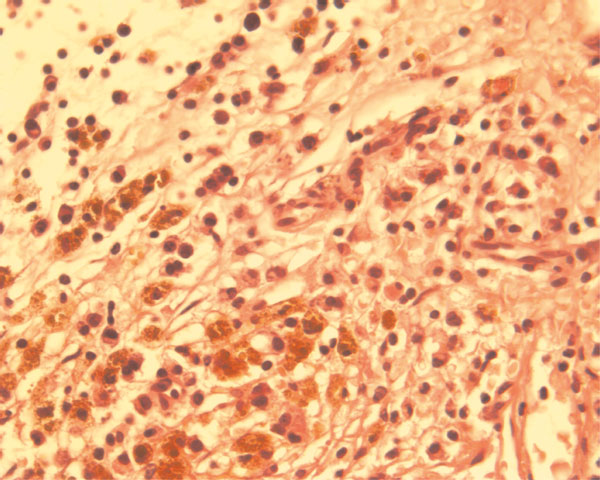
**Topical hemosiderin pigmentation of the chorium expressed the catabolism of haemoglobin structure due to micro-hemorrhages (H&E ×20)**.

The decalcified tooth sections showed the presence of dystrophic calcification into the canal with peripheral reactionary dentin against the root (Figure 4). The apical part was obstructed due to excessive formation of reactionary dentin. Bacterial clouds were identified with necrotic organic contents and possible haem catabolism. The bacterial invasion of dentinal tubules was a common pattern. Also, foam cells, fibrosis and topical hemosiderin pigmentation (Figure 5) were identified in the chorium of the radicular cyst.

## Discussion

Radicular cysts are common osseo-destructive jaw lesions. Uncommon microscopical features of radicular cysts include subepithelial fibrosis, topical hemosiderosis and lipid-laden macrophages in association with a root canal dystrophic calcification.

Root canal dystrophic calcification in aggressive forms may cause obliteration of the root canal apex [9]. In most of the cases, liquefaction foci can be identified in the canal or overlying the dentin. The presence of liquefaction products in the canal indicates chronicity as these are usually produced from the detachment of the peripheral dentin. This condition is very difficult to treat by conventional methods (i.e. lege artis endodontic treatment). Surgical excision of the periapical lesion followed by apicectomy is considered to be the treatment of choice.

Lipid-laden macrophages or foam cells are derived from the monocyte system and take part in removing the necrotic tissue [7]. The phagocytosis of the lipid debris induces microscopic expansion of the macrophages; this usually appear as parenchymal round or oval empty spaces [10]. This host-defense mechanism shows the difficulty in digesting lipids. The presence of neutrophils (PMN's) may be indicative of the current symptoms. Acute exacerbation of a radicular cyst may be characterized by plethora of PMN's in the field of chronicity.

Another form of chronic irritability may be identified by the presence of subepithelial fibrosis. This is clearly associated with fibroblast stimulation creating fibrotic islands in the chorium [11]. This appears as scar tissue formation when there is inability of the host-defense mechanism to restore physiological function. Therefore subepithelial scar formation is a process of chronically destructive stimuli.

Other rare forms of chronicity may be the hemosiderin pigmentation which is related to the deposition of endogenous catabolic products. In cases where hemosiderin is found in multiple internal organs, the term "systemic hemosiderosis" seems to be more appropriate. The hemosiderin pigmentation indicates the presence of many micro-hemorrhages in the lamina propria. Cells of the defense mechanism (i.e. macrophages) react at the site of erythrocyte degradation; this starts "phagocytosis" [6]. Hemosiderin excess may be observed on light microscopy especially if Prussian-blue is used in histopathological staining [12]. In our case, H&E stain was used to investigate the presence of this catabolic pigmentation.

This case report described the association of pulp dystrophic calcification with subepithelial fibrosis, lipid laden macrophages and hemosiderin pigments on the chorium of a radicular cyst. In conclusion, the synchronous existence of these pathological features in a radicular cyst may indicate the development of chronic irritation in periodontal tissues by the root canal microbial proliferation.

## Abbreviations

EDTA: ethylenediaminetetraacetic acid; H&E: hematoxylin & eosin; PMN's: polymorphonuclear neutrophils.

## Consent

"Written informed consent was obtained from the patient for publication of this case report and accompanying images. A copy of the written consent is available for review by the Editor-in-Chief of this journal."

## Competing interests

The authors declare that they have no competing interests.

## Authors' contributions

PK, SK, WJ, TU were major contributors in assessing the case data, reviewing and writing the manuscript. All authors read and approved the final manuscript.
